# CorA Is a Copper Repressible Surface-Associated Copper(I)-Binding Protein Produced in *Methylomicrobium album* BG8

**DOI:** 10.1371/journal.pone.0087750

**Published:** 2014-02-03

**Authors:** Kenneth A. Johnson, Thomas Ve, Øivind Larsen, Rolf B. Pedersen, Johan R. Lillehaug, Harald B. Jensen, Ronny Helland, Odd A. Karlsen

**Affiliations:** 1 Norwegian Structural Biology Centre, Faculty of Science, University of Tromsø, Tromsø, Norway; 2 Department of Molecular Biology, University of Bergen, Bergen, Norway; 3 Department of Earth Science - Centre for Geobiology, University of Bergen, Bergen, Norway; Saint Louis University, United States of America

## Abstract

CorA is a copper repressible protein previously identified in the methanotrophic bacterium *Methylomicrobium album* BG8. In this work, we demonstrate that CorA is located on the cell surface and binds one copper ion per protein molecule, which, based on X-ray Absorption Near Edge Structure analysis, is in the reduced state (Cu(I)). The structure of endogenously expressed CorA was solved using X-ray crystallography. The 1.6 Å three-dimensional structure confirmed the binding of copper and revealed that the copper atom was coordinated in a mononuclear binding site defined by two histidines, one water molecule, and the tryptophan metabolite, kynurenine. This arrangement of the copper-binding site is similar to that of its homologous protein MopE* from *Metylococcus capsulatus* Bath, confirming the importance of kynurenine for copper binding in these proteins. Our findings show that CorA has an overall fold similar to MopE, including the unique copper(I)-binding site and most of the secondary structure elements. We suggest that CorA plays a role in the *M. album* BG8 copper acquisition.

## Introduction

Copper plays an important role in methane oxidizing bacteria and is directly involved in controlling methanotrophic activity [1], [2], [3]. In Type II methanotrophs, which possess two versions of the methane monooxygenase (MMO), the copper-to-biomass ratio regulates the expression of the particulate membrane bound MMO (pMMO) and the soluble cytoplasmic MMO (sMMO) [2], [4], [5]. In cells expressing pMMO (including both Type I and Type II methanotrophs), it has been demonstrated that copper can further stimulate pMMO expression and alter both its substrate affinity and specificity [6], [7]. The availability of copper also regulates the formation of a complex network of intracytoplasmic membranes at which the oxidation of methane by pMMO takes place [8]. In the model methanotroph, *Methylococcus capsulatus* Bath, it has been extensively demonstrated that the availability of copper has significant impact on the proteome, including the expression of several outer membrane and surface-associated proteins, hemerythrin, and at least two formaldehyde dehydrogenases [9], [10], [11], [12], [13], [14], [15], [16]. However, considering the importance of copper in the physiology of methanotrophs, the mechanism of its acquisition by the bacteria is to a large extent unknown. Copper-binding chalkophores, denoted methanobactins, has been shown to be important for handling and transport of copper into several methanotrophic bacteria [3], [17], but recent findings suggest that some methanotrophs utilize a surface located protein of great importance for the same purpose [10], [18], [19], [20].

*Methylomicrobium album* BG8 is a Type I methanotroph that solely uses pMMO for the initial oxidation of methane [21]. pMMO is a copper-containing enzyme and needs both reduced (Cu(I)) and oxidized (Cu(II)) copper for its enzymatic activity [22]. Distinct from the switch-over methanotrophs (Type II) that can produce sMMO at low copper-to-biomass regimes, *M. album* BG8 is highly dependent on the availability of copper ions for growth and efficient oxidation of methane. A copper repressible protein, denoted CorA, was previously identified from a particulate fraction of *M. album* BG8 cells [23]. CorA shares significant sequence similarity to the *M. capsulatus* Bath protein MopE and the recently described MEALZv2_1030034 protein isolated from *Methylomicrobium alcaliphilum* 20Z (Fig. S1) [20], [24]. CorA is smaller compared to MopE, and the sequence similarity is therefore restricted to the MopE C-terminal part, i.e. the secreted domain denoted MopE* [24]. Like CorA, the synthesis of MopE and MEALZv2_1030034 are repressed by copper ions, and it was recently demonstrated that MopE binds copper [18], [20], [25]. The crystal structure of MopE* revealed that one of the copper binding ligands is the tryptophan metabolite kynurenine [18]. This was the first report of the involvement of kynurenine as a metal coordinating ligand in a protein. Interestingly, all of the copper coordinating ligands, including the two histidines and the tryptophan kynurenine-precursor, are conserved residues between MopE* and CorA (Fig. S1) (ibid.). However, the copper binding capabilities of CorA has remained unknown. Importantly, a constructed *M. album* BG8 *corA* knock-out mutant grew very poorly and was not able to sustain growth even at higher copper concentrations (10 µM), implying that CorA is essential for growth [23]. It was therefore proposed that CorA has a role in the handling and transport of copper into the cells, which has received recent support by biochemical studies indicating that the homologous proteins, MopE and MEALZv2_1030034 have similar roles in copper handling [20], [25].

In the present work, we show that CorA is non-covalently associated to the *M. album* BG8 outer membrane and exposed on the cellular surface, contrary to the previously assumed localization to the Gram-negative inner membranes [23]. Furthermore, both endogenously- and recombinantly expressed CorA were purified to homogeneity, and inductively coupled plasma mass spectrometry (ICP-MS) analyses demonstrated that only endogenously expressed CorA binds copper, one atom per molecule. X-ray Absorption Near Edge Structure analysis indicated that this copper is in the reduced state (Cu(I)). Furthermore, X-ray crystallography was used to solve the structure of CorA, and provided detailed information on its copper-binding site. The CorA copper-binding site closely resembles the mononuclear binding site present in MopE*, i.e. the copper atom is coordinated by two histidines, one water molecule, and the tryptophan metabolite kynurenine [18].

## Materials and Methods

### Growth of *Methylomicrobium album* BG8

*M. album* BG8 (NCIMB 11123^T^) was grown in 100 ml batch cultures at 30°C while shaking, in nitrate mineral salt medium and an atmosphere of methane and air (50∶50) [26]. Cells were grown either at a low copper-to-biomass ratio where 0.1 µM copper was added to the growth medium (low copper grown cells), or at a high copper-to-biomass ratio with 5 µM copper added to the medium (high copper grown cells). For protein purification of CorA, *M. album* BG8 was cultured in a 5 L fed-batch fermentor. A 100 ml culture was used as an inoculum to 4.9 L NMS medium containing 0.2 µM CuSO_4_ in a L1523 fermentor (Bioengineering, Switzerland). The fermentation was performed at 30°C under an atmosphere of air-methane with oxygen as the limited nutrient. The pH was continuously adjusted to 7.0 by automatic addition of 0.5 M HNO_3_. After reaching a cell density of OD_600__nm_ = 4.5 the culture was harvested by centrifugation (10,000 *g* for 30 min).

### Sodium dodecyl-polyacrylamide gel electrophoresis (SDS-PAGE) and Western blotting

SDS-PAGE was performed as described by Laemmli [27]. Western blotting was carried out as described previously [28].

### Cell fractionation

Cells were harvested by centrifugation at 5000 *g* for 10 min (100 ml batch cultures), or by centrifugation at 10,000 *g* for 30 min (fermentor culture). Fractions enriched in soluble proteins, inner-membrane proteins, and outer-membrane proteins were obtained as described previously by Karlsen et al. [28], [29].

### Extraction of surface- and outer-membrane-associated proteins

Cell-surface-associated proteins and outer-membrane-associated proteins were extracted with NaCl as described previously [28], [29].

### Mass spectrometry analyses

Matrix-associated laser desorption/ionization mass spectrometry analyses were performed as described previously using an Ultraflex 2 mass spectrometer [13]. Overnight protease “in-gel” digestion of CorA, and reduction/alkylation of cysteines were carried out essentially as described by Laugesen and Roepstorff and Schevchenko et al., respectively [30], [31]. LTQ-ORBITRAP analyses were performed at the Mass Spectrometry Service Facility at the University of Oslo, Norway. The copper and sulphur content of CorA were determined by Inductively Coupled Plasma Mass Spectrometry (ICP-MS) at the Center for Element and Isotope Analyses (CEIA), University of Bergen, Norway. Prior to analysis, the samples were hydrolysed overnight with nitric acid (6% v/v) at 110°C. A single collector double focusing magnetic sector field ICP-MS spectrometer (Finnigan Element 2) was used for the copper and sulphur analyses. The samples were diluted in 2% HNO_3_ and analysed by the standard addition method using an ICP multi-element standard (Merck # 1.0580.0100) for calibration. Oyster Tissue (NIST 1566a) was used as an external reference standard.

### Purification of endogenous and recombinantly expressed CorA

Triton X-100 insoluble membranes obtained from fermentor-grown *M. album* BG8 cells were washed with 20 mM Tris-HCl pH 7.5, 1 mM CaCl_2_ and 1 M NaCl with rotation for 1 h at 4°C. Insoluble components of the CorA-containing extract were removed by centrifugation at 100,000 *g* at 4°C for 1 h. The resulting supernatant containing CorA was desalted using PD10 columns equilibrated with 40 mM Tris-HCl, pH 8.0. Contaminating proteins were further removed by precipitation using 50% saturated (NH_4_)_2_SO_4_ (2.05 M) for 1 h at room temperature. Precipitated material was removed by centrifugation at 10.000 *g* for 10 min, and (NH_4_)_2_SO_4_ was removed from the CorA-containing supernatant by de-salting on PD10 columns using the 40 mM Tris-HCl, pH 8.0 solution. The protein was then loaded onto a 1 ml DEAE FF (GE Healthcare) anion exchanger (flow 0.2 ml/min) and CorA was eluted with a 20 ml linear NaCl gradient (40 mM Tris-HCl pH 8.0, and 0–0.3 M NaCl). Fractions containing CorA were pooled and subjected to Superdex 75 16/60 (GE Healthcare) gel filtration (40 mM Tris-HCl pH 8.0, 0.5 M NaCl). Homogenous CorA fractions were collected, pooled, and stored at 4°C until use. Typically, Triton X-100 insoluble membranes obtained from 1.2 ml of *M. album* BG8 wet-pellet was used as the starting point for the purification procedure outlined above, which based on the CorA molar extinction coefficient (42890 M^−1^ cm^−1^) and absorbance measurement at 280 nm, yielded approximately 0.3 mg of purified endogenous CorA. Recombinant CorA (without the predicted signal peptide) was expressed in *E. coli* BL21 (DE3) as an MBP (maltose binding protein) fusion protein containing an N- terminal hexahistidine tag and a TEV protease recognition site using the pETM41 vector (provided by G. Stier). Cultures were grown at 37°C in LB broth to an A_600 nm_ of ≈0.6–0.8, and expression was induced by adding 1 mM isopropyl thio-β-d-galactoside. After overnight incubation (16–18 h) at 18°C, cells were harvested by centrifugation at 5,000 *g* for 10 min at 4°C and resuspended in 50 mM Tris-HCl pH 7.5, 0.1 M NaCl, 0.4 mM AEBSF-hydrochloride (Applichem), and 10 mM imidazole. The re-suspended cells were lysed by sonication and the lysate clarified by centrifugation at 15,000 *g* for 30 min at 4°C. The soluble fraction was loaded onto a 5 ml HisTrapTM chelating column (GE Healthcare) and bound proteins were eluted by a linear gradient of imidazole (10 – 250 mM) in 50 mM Tris-HCl pH 7.5 and 0.5 M NaCl. Fractions containing the MBP–CorA fusion protein were pooled and run on a Superdex 75 16/60 (GE Healthcare) gel filtration column. MBP and the hexahistidine tag were removed by overnight incubation with the TEV protease (Invitrogen, Carlsbad, CA, USA) treatment, and cleaved CorA was further purified using a HisTrapTM chelating column, followed by a final gel filtration (Superdex 75 16/60) in 20 mM Tris-HCl pH 7.5, 1 mM CaCl_2_. The yield of rec-CorA purified from 1 L of bacterial culture was approximately 2–3 mg (based on the CorA molar extinction coefficient and absorbance at 280 nm).

### Circular dicroism

CD spectra of recombinant CorA were recorded on a Jasco J-810 spectra-polarimeter at 25°C. Prior to analysis, the protein was dialyzed against 10 mM potassium phosphate buffer pH 7.5, and diluted to a final concentration of 15 µM. Measurements were recorded at 0.5 nm wavelength increments from 260 to 200 nm at 50 nm/min, using a 0.1 mm path length cell, 0.5 nm band-width, 1 s response time, and four accumulations. The spectra were corrected for buffer base line contribution, and analyzed by the programs K2D2, ContinII, Selcon3 and CDSSTR [32], [33].

### Crystallization, data collection, structure determination and analysis

Initial crystallization trials were set up using an Art Robbins Phoenix crystallization robot to create 96-well crystallization setups using 60 µl in the reservoirs and 100 nl protein solution plus 100 nl reservoir solution in the experimental drops. Crystals were obtained from conditions in 3 homemade stochastic screens and optimized in 24-well hanging drop plates set up by hand [34]. Crystals suitable for data collection were obtained using a CorA protein solution of 4 mg/ml, and reservoir solutions containing 11.5% to 14% polyethylene glycol 8000 and 0.1 M BisTris, pH 7.0. For data collection, crystals were cryo-protected with a solution containing 16% polyethylene glycol 8000, 0.1 M BisTris, pH 7.0 and 24% polyethylene glycol 400. Heavy atom-soaked crystals were prepared by soaking a native crystal in the cryo-protecting solution diluted 1∶10 with various heavy atom-containing stock solutions. A successful derivative was obtained by soaking a crystal in 20 mM K_3_IrCl_6_.

The K_3_IrCl_6_ phasing data set to 1.85 Å was collected on a Rigaku Raxis IV++ detector system, and the native data set to 1.6 Å was collected at beamline ID29 (European Synchrotron Radiation Facility, Grenoble (ESRF) (Table 1). Data were processed using XDS and scaled using XSCALE [35]. Heavy atom sites were found by SOLVE [36] and phases improved by RESOLVE [37] using data from the K_3_IrCl_6_-soaked crystal to 3.3 Å. Six molecules of a trimmed, polyalanine model made from the homologous MopE* structure (PDB entry 2VOV, [18]) were located in this initial experimental electron density map by molecular replacement [38]. A selected set of coordinates from this model was used to automatically generate 6-fold non-crystallographic symmetry (Fig. S2) restraints for phase improvement at 2.8 Å producing an experimental map that was used to automatically build a model containing 900 amino acid residues [39]. After refinement with REFMAC5 [40], the model was used as input to ArpWarp [41] at 1.94 Å using a home data set (data not shown) and resulting in a model containing 1100 residues that was corrected by hand using XTALVIEW [42] and Coot [43] and refined using REFMAC5 iteratively. This yielded an almost complete model containing 1230 residues that was completed using a 1.6 Å native data set (Table 1). The structure has been deposited in the PDB with reference number 4BZ4. The structure based sequence alignment of CorA, MopE* and MEALZv2_1030034 (Fig. S1) was generated by combining a structural alignment of CorA and MopE* generated by the Dali server (http://ekhidna.biocenter.helsinki.fi/dali_lite/) [44], and a standard sequence alignment of CorA and MEALZv2_1030034 using Clustal X.. Illustrations of the 3D structure were prepared using PyMOL (http://www.pymol.org) and electrostatic surface potentials were generated using the APBS plugin (Adaptive Poisson-Boltzmann Solver) [45].

**Table 1:** Crystal Parameters, X-ray Data Collection, and Refinement Statistics.

	Native	K_3_IrCl_6_ soak
Crystal Parameters	ESRF, ID29	Home, RAXIS IV++
Space group	P2_1_	P2_1_
Unit cell edges(Å) and β-angle(°)	a = 73.8,b = 113.2,c = 81.5,β = 104.5	a = 73.8,b = 113.5,c = 82.0,β = 104.5
Data collection statistics		
Wavelength (Å)	0.9794	1.5418
Resolution range (Å)^a^	30 – 1.6 (1.64 – 1.6)	30 – 1.85 (1.89 – 1.85)
No. of unique reflections	168264 (12631)	213133 (14459)
Completeness (%)	98.6 (97.0)	96.3 (88.4)
R_merge_ (%)^b^	4.9 (42.9)	5.5 (57.7)
Redundancy	2.8 (2.7)	8.3 (7.9)
Mean I/sigma(I)	15.3 (2.8)	27.3 (4.1)
Refinement and model statistics		
Resolution range(Å)	30 – 1.6 (1.64 – 1.6)	
R_work_ (%)^c^	15.8 (23.9)	
R_test_ (%)^c^	18.8 (27.1)	
RMSD bonds (Å)	0.02	
RMSD angles (°)	2.01	
DPI^d^	0.076	
Validation by MOLPROBITY		
Ramachandran favored (%)	97.36	
Ramachandran outliers (%)	0	
Rotamer outliers (%)	1.05	
PDB code	4BZ4	

a The values in parentheses are for the highest resolution shell.

b *R*_merge_ = ∑*hkl* ∑*j* |*Ij*−*I* |/∑*hkl*∑*j Ij* where *Ij* is the intensity from an individual measurement of reflection *hkl* and *I* is the mean intensity of the same reflection.

c *R*_work_ = ∑*hkl* |*F*o−*F*c|/∑*hkl* |*F*o|; *R*_test_ is the same but for the 5% of the total reflections never used for refinement.

d Cruickshanks DPI for coordinate error.

DPI = sqrt(N_atom_/(N_refl_−N_param_)) R_factor_ D_max_ compl^−1/3^. N_atom_ is the number of the atoms included in the refinement, N_refl_ is the number of reflections included in the refinement, R_factor_ is the overall R-factor, D_max_ is the maximum resolution of reflections included in the refinement, compl is the completeness of the observed data.

### X-ray Absorption Near Edge Structure (XANES)

The XANES spectrum of native cryo-cooled CorA crystals was recorded at beamline ID29 (ESRF) independent from X-ray data collection. The spectrum was recorded in steps of 0.7 eV while measuring counts over the copper K edge, from 8940 eV to 9060 eV.

## Results

### Identification and cellular localization of CorA

**Figure 1:**
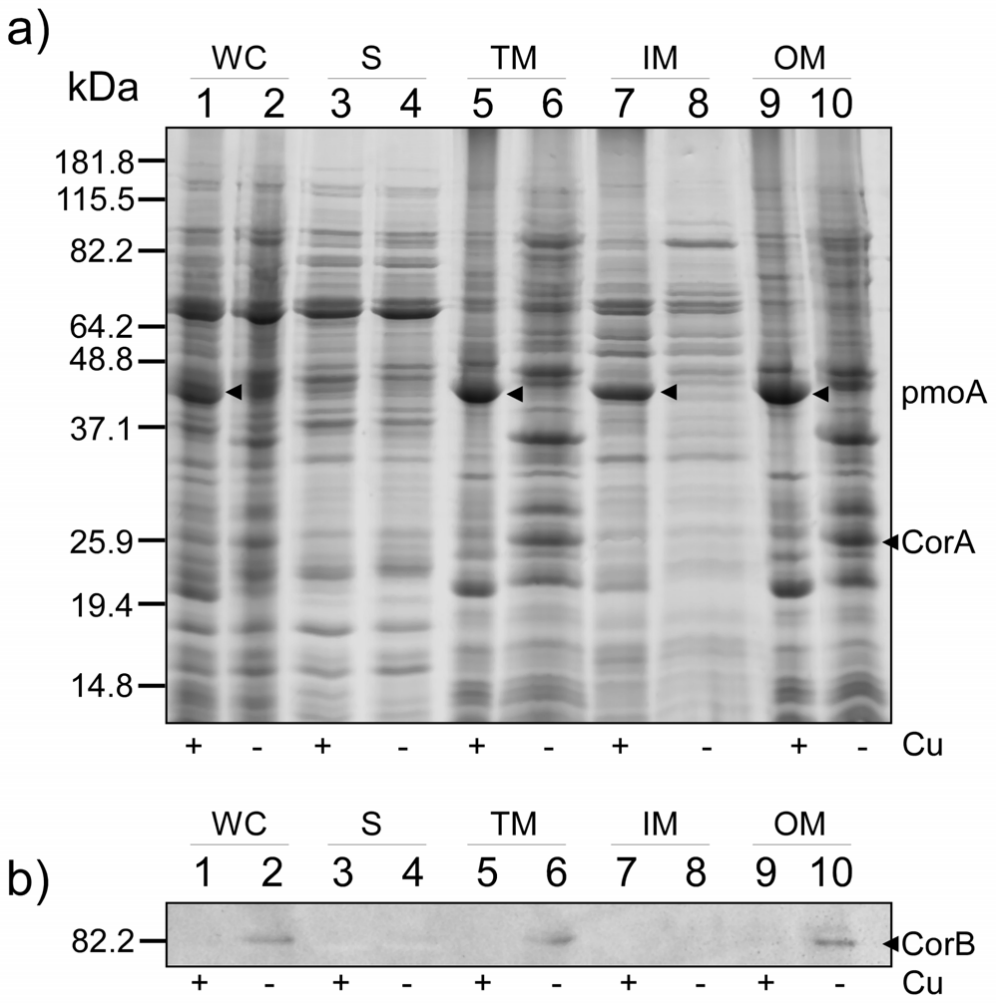
SDS-PAGE and protein immunoblot analysis of proteins obtained during the fractionation of high- and low-copper grown *M. album* BG8. Samples from each fractionation step were collected and comparable amounts were analyzed. a) A 12.5% PA-gel was used and stained with Coomassie Brilliant Blue R-250. a) and b) Lane 1 and 2, whole cells (WC); lane 3 and 4, soluble fraction (S); lane 5 and 6, total membrane fraction (TM); lane 7 and 8, Triton X-100 soluble membrane fraction (enriched inner-membrane fraction, IM); lane 9 and 10, Triton X-100 insoluble fraction (enriched outer membrane fraction, OM). High- (+) and low-copper (−) conditions during growth are indicated below the PA-gel. b) Protein immunoblot of a) using CorB-specific antibody [28]. CorA and putatively the pmoA subunit of the *M. album* BG8 pMMO are indicated with arrowheads. Molecular mass markers are indicated to the left of both a) and b).

**Figure 2:**
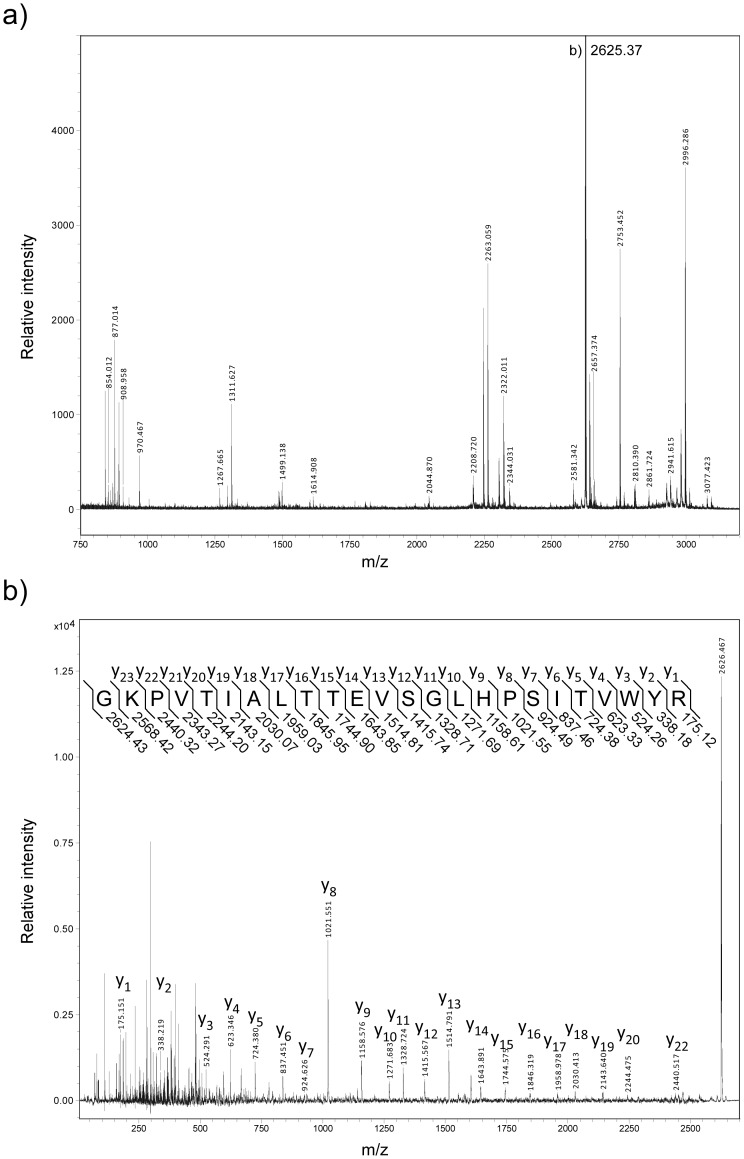
MALDI-TOF MS and MS/MS analyses of the PA-gel excised 26 kDa polypeptide. a) MALDI-TOF MS spectrum of trypsin-produced peptides derived from the 26 kDa polypeptide excised from the outer membrane fraction of low-copper grown *M*. *album* BG8. Monoistopic peaks are labeled with their respective *m/z* ratio. b) MALDI-TOF MS/MS spectrum of the *m/z* 2625.37 ion, indicating the observed fragmentation pattern and the sequence ion assignment.

**Figure 3:**
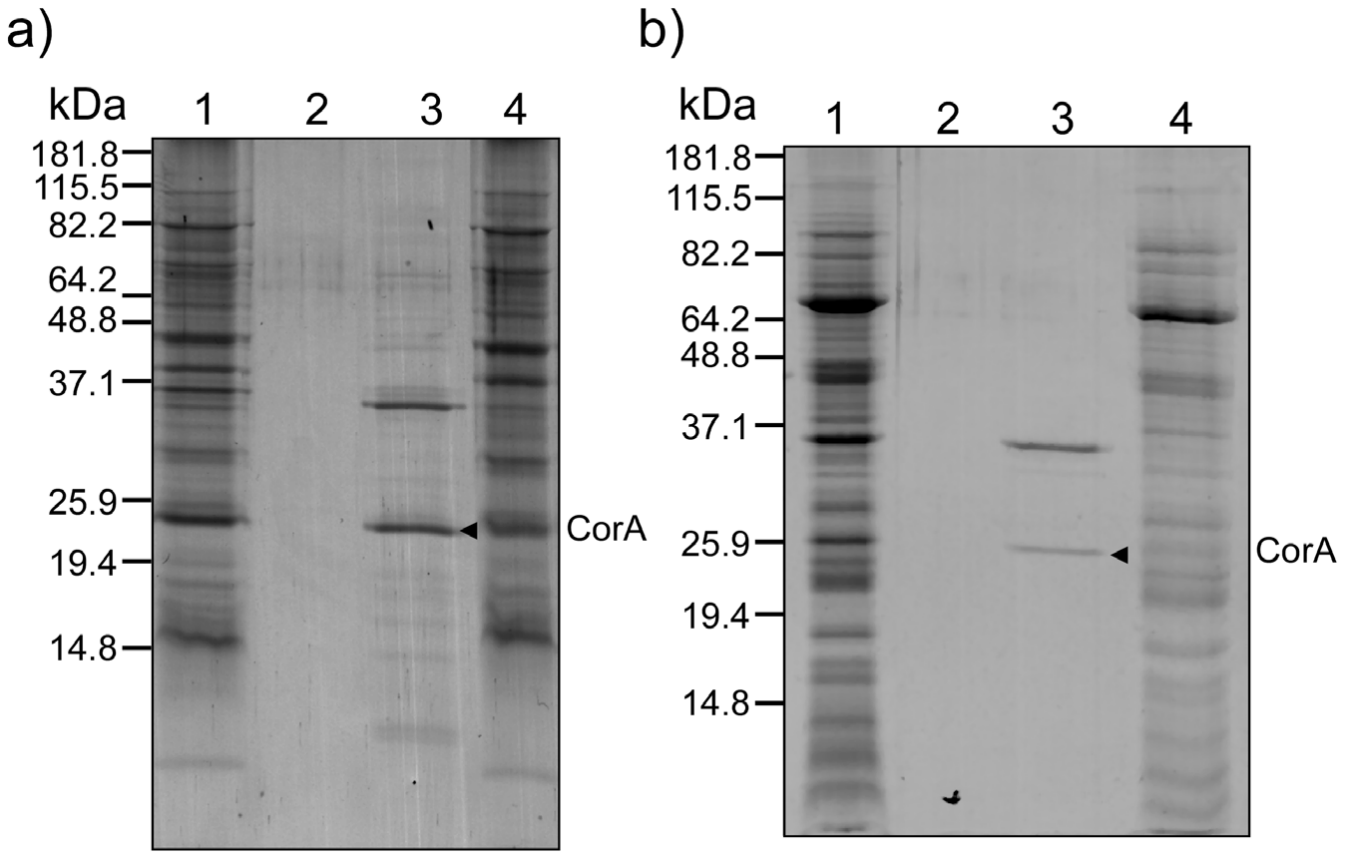
SDS-PAGE analyses of protein fractions obtained from NaCl extraction of a) the outer membrane and b) of whole fermentor-grown cells. 12.5% PA-gels were used and stained with Coomassie Brilliant Blue R-250. a) Lane 1, *M. album* BG8 outer membrane fraction; lane 2, Tris-HCl wash of low ionic strength; lane 3, 1 M NaCl extract of the outer membrane fraction; lane 4, outer membrane fraction after treatment with 1 M NaCl. b) Lane 1, *M. album* BG8 cells resuspended in buffer of low ionic strength; lane 2, Tris-HCl wash of low ionic strength; lane 3, 1 M NaCl extract of whole cells; lane 4, whole cells after treatment with 1 M NaCl. CorA is indicated in both a) and b). Molecular mass markers are indicated.

To explore the cellular localization of CorA, low- and high-copper grown *M. album* BG8 cells were fractionated using the protocol previously applied to both *M. capsulatus* Bath and *M. album* BG8 (M&M and [28], [29]). The protein composition in the resulting fractions, including the soluble fraction (cytoplasm and periplasm), the Triton X-100 soluble fraction (inner membranes), and the Triton X-100 insoluble fraction (outer membranes) were assessed by SDS-PAGE (Fig. 1A). Several differences in the protein profiles were revealed between high- and low-copper grown cells. This was especially evident in the membrane fractions, including the higher abundance of polypeptides presumably corresponding to subunits of pMMO in the high-copper grown cells (indicated in Fig. 1a). A corresponding protein immunoblot using antibodies recognizing the CorA co-transcribed c-type heme protein, CorB, showed, in line with previous data, the copper-regulated synthesis of CorB and confirmed that the *corA-corB* operon was transcribed under the low copper-to-biomass growth condition (Fig. 1b) [28]. Inspection of the Triton X-100 insoluble fractions from the low-copper grown cells revealed an abundant copper-repressible polypeptide of approximately 26 kDa (Fig. 1a). Its molecular mass is slightly higher than the predicted theoretical mass of CorA, but in line with the electrophoretic migration of CorA on SDS-PA gels reported previously [23]. This polypeptide band was excised from the polyacrylamide gel, digested with trypsin, and analyzed using MALDI-TOF MS/MS mass spectrometry (Fig 2). Database searches (NCBInr) using the resulting MS and MS/MS data showed a significant match against the *M. album* BG8 CorA protein (Ac. no: AAC45111) mapping 8 peptides to its amino acid sequence with 35% sequence coverage. Furthermore, the recorded MS/MS spectrum revealed a near complete y-ion series derived from a 2625.37 Da internal CorA peptide (G76-R99), verifying that the excised polypeptide represented the CorA protein (Fig. 2b). Importantly, the presence of CorA in the Triton X-100 insoluble fraction indicates that CorA is located to the *M. album* BG8 outer membrane. To further determine the cellular localization of CorA, we carefully washed both the Triton X-100 insoluble fraction and whole cells (fermentor grown cells, see M&M) with buffer of high ionic strength (1 M NaCl) to extract non-covalently associated proteins from the outer membrane and the cellular surface, respectively. SDS-PAGE analyses showed that CorA was not retained on the outer membrane (Fig 3a), or on cells (Fig 3b) following treatment with high-salt buffer, but the protein was recovered in the high-salt fractions. These results demonstrate that CorA is non-covalently associated to the *M. album* BG8 outer membrane on the cellular surface. In contrast to the *M. capsulatus* Bath MopE*, CorA was not identified in the spent medium of *M. album* BG8.

### Purification and copper-binding properties of CorA and recombinant CorA

**Figure 4:**
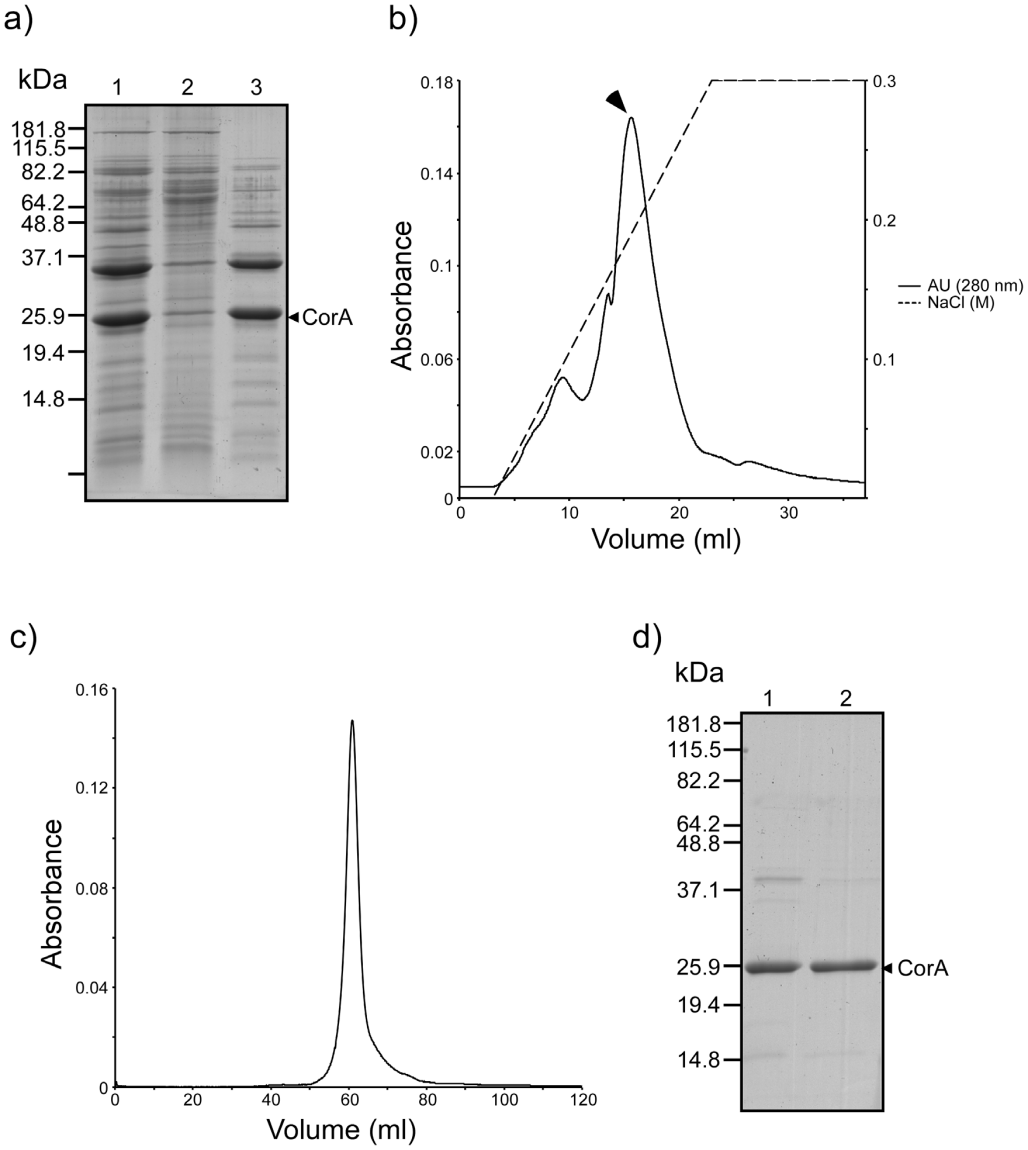
Purification of endogenously expressed CorA. a) SDS-PAGE analysis of fractions obtained after the (NH_4_)_2_SO_4_ precipitation of the CorA-containing NaCl extract (see M&M). A 12.5% PA-gel was used and stained with Coomassie Brilliant Blue R-250. Lane 1, PD10 desalted NaCl extract from the *M. album* BG8 outer membrane; lane 2, solubilized precipitated material after 50% (NH_4_)_2_SO_4_ precipitation of 1); lane 3, The soluble fraction after 50% (NH_4_)_2_SO_4_ precipitation of 1). b) A representative chromatogram of the anion exchanger elution profile of the (NH_4_)_2_SO_4_-treated NaCl extract. Elution of CorA is indicated with an arrowhead. c) A representative gel filtration chromatogram of pooled CorA-containing fractions obtained in the anion exchanger chromatography. d) SDS-PAGE analysis of pooled fractions containing CorA obtained from the ion exchanger chromatography (lane 1), and the gel filtration (lane 2). CorA and molecular mass markers are indicated.

**Figure 5:**
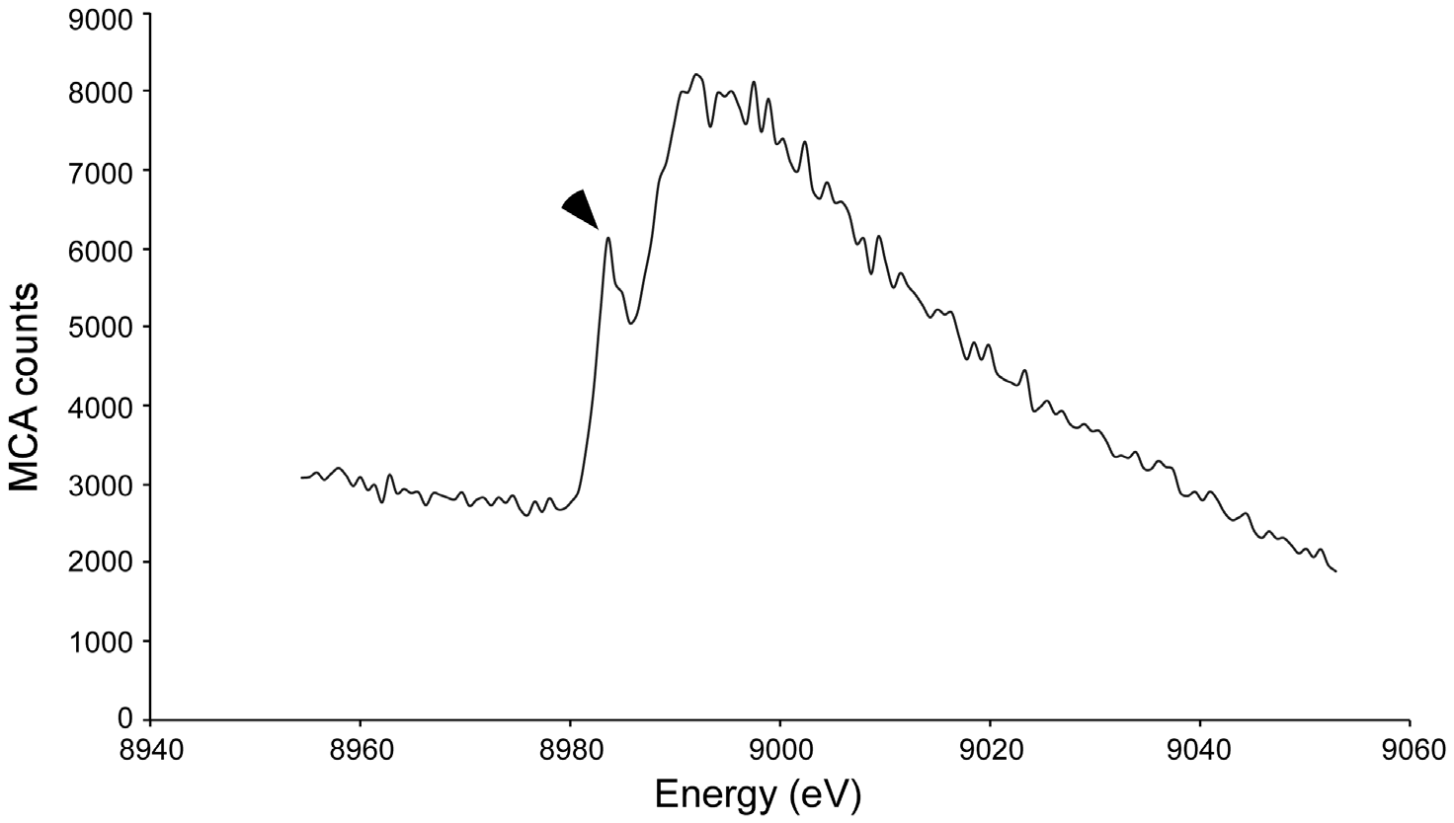
X-ray Absorption Near Edge Structure analysis of endogenously expressed CorA. XANES spectrum of crystalline CorA, recorded in steps of 0.7-edge feature at 8984 eV, typical for Cu(I) compounds (marked with an arrowhead).

In order to explore the copper-binding capabilities of CorA, both endogenous and recombinantly expressed CorA (rec-CorA) were purified to homogeneity (M&M). In order to avoid contamination by intracellular and periplasmic proteins resulting from cell lysis when treating large amounts of cells with buffer of high ionic strength, we chose to use Triton X-100 insoluble membrane (outer membrane) fractions as the starting point for the high salt extraction of CorA from the *M. album* BG8 (Fig. 3a). The CorA-containing fraction was made 50% in ammonium sulphate to remove several contaminating protein species (Fig. 4a). Homogenous CorA fractions of high purity were then obtained by anion exchange chromatography followed by size exclusion chromatography (Fig. 4b,c,d, see M&M). The retention time of CorA (and of rec-CorA) on the calibrated gel filtration column corresponded to a molecular mass approximately twice the monomeric mass of CorA, indicating that CorA may exist as a dimer *in vivo* (data not shown). Rec-CorA was exogenously expressed in *Escherichia coli* BL21, and purified as described previously for rec-MopE* (Fig. S3, see M&M) [18]. A circular dichroism (CD) spectrum (Fig. S4) recorded of rec-CorA verified that the *E. coli*-expressed protein was recovered in a folded conformation, and the negative minimum at 217 nm is consistent with the presence of predominant β-sheet structure. The copper content of CorA and rec-CorA was determined by ICP-MS (M&M), showing that endogenous type CorA isolated from *M. album* BG8 contained one copper atom per protein molecule, while no copper could be detected in rec-CorA expressed in *E. coli.* This observation is in line with ICP-MS data obtained for the copper content of MopE* and rec-MopE* [18]. We then used XANES to examine the oxidation state of the CorA-bound copper. The XANES spectrum (Fig. 5) showed a distinct pre-edge feature at 8983-8984 eV attributed to the 1s→4p transition typical for reduced copper (Cu(I)) complexes. This feature is not observed for Cu(II) complexes [46]. The spectrum thus suggests that the CorA-bound copper is in the reduced (Cu(I)) state, which is in agreement with data obtained for MopE* [25]. The position of the feature is comparable to those reported previously for Cu(I) centres in methane monooxygenase (8983–84 eV) [22] and Cu^+^-ATPases (8984 eV) [47].

### Crystal structure of the CorA copper-binding site

Sequence alignments revealed that the MopE* copper-coordinating ligands are conserved in CorA and MEALZv2_1030034 (Fig. S1), including the tryptophan oxidized to kynurenine in MopE*. However, neither CorA, nor rec-CorA, produced a proteolytic fragment (using either trypsin, chymotrypsin or trypsin/CNBr combination,) containing either Trp(62) or Kyn(62) that could be recovered in MALDI-TOF, or LTQ-ORBITRAP mass spectrometry analyses (Data not shown). Thus, in order to obtain further information on the CorA copper-binding site, we produced sufficient amounts of endogenous CorA protein to grow crystals for X-ray diffraction analysis. Native CorA crystals diffracted to a resolution of 1.6 Å. There were six molecules in the asymmetric unit, all having well defined electron density for most of the molecule, with the exception of a few surface loops

**Figure 6:**
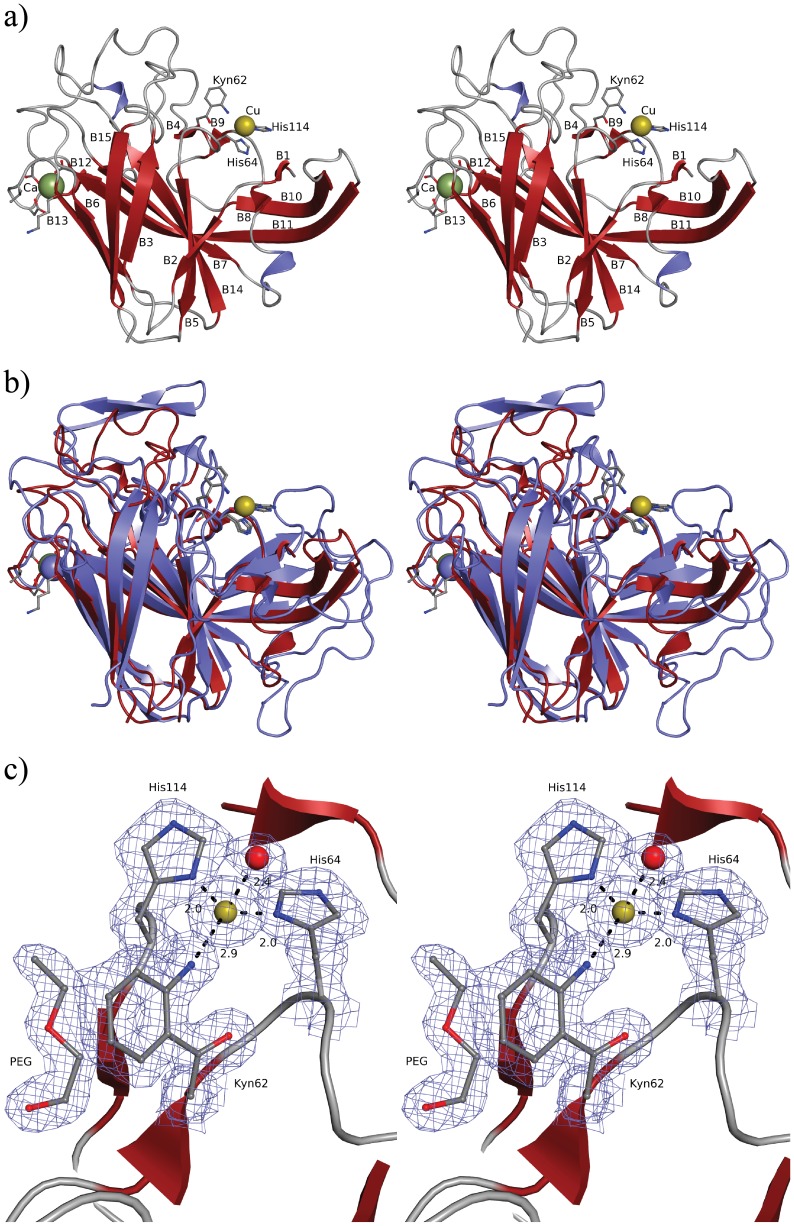
Overall view and copper-binding site of CorA. a) Stereo plot of CorA, illustrating the 15 β-strands, Cu(I)(yellow sphere), Ca^2+^ (green sphere), and residues coordinating the metal ions (ball-and-stick models). b) Superimposition of CorA (red) and MopE* (blue). The copper and calcium in CorA are illustrated as yellow and green spheres, respectively. The metals in MopE* are coloured blue. Because of the superposition calcium appears as blue and copper as yellow. The 15 β-strands of CorA superimpose well on 15 of the 21 strands found in MopE*. Also the residues involved in copper and calcium binding superimpose well. c) Stereo plot illustrating the 2foDfc electron density, contoured at 1 σ in the copper binding site, demonstrating the oxidation of tryptophan to kynurenine at residue 62. The copper and the coordinating water are illustrated as yellow and red spheres, respectively. The polyethylene glycol molecule shown is found near the kynurenine residue in all CorA molecules in the crystal structure.

Consistent with the CD spectrum of rec-CorA, the structure (Fig. 6a) contains mainly β-strands forming two β-sheets and random coils and very little helical content. CorA contains two metal ions, which by analogy to MopE* was interpreted as calcium and copper, and the latter confirmed by ICP-MS. The 15 β-strands of CorA superimpose well on secondary structure elements determined for MopE* (Figs. 6b and S1). The six CorA molecules superimposes on MopE* with root mean squared deviation (rmsd; [48]) values in the range from 1.4 Å to 1.8 Å for 154 to 163 superposed residues. The seven CorA residues involved in the binding of the two metals (Cu and Ca) exhibit much lower rmsd values, ranging from 0.3 Å to 0.8 Å, indicating that the respective metal-binding regions of the MopE* and CorA structures are more structurally conserved than the overall structures. In accordance, the coordination geometry of the two metal ions in the CorA structure (Fig. 6a), is very similar to those of the metal ions in the MopE* structure (Fig 6b) [18]. The electron density clearly shows that the copper ion is, as in MopE*, coordinated by two histidines (H64 and H114), the tryptophan metabolite kynurenine (Kyn62), and a water molecule (Fig. 6c)). The geometry is distorted tetrahedral with the copper ion almost at the base of the pyramid formed by residues Kyn62, H64 and H114 and the solvent molecule at the apical position (Fig. 6b) displaying geometrical similarities to Type I (blue copper proteins) and Cu_B_ copper centres [49], [50]. The two histidine ND1 atoms form a near tetrahedral angle of 105.9±1.9° with metal-ligand bond distances of about 2.05±0.08 Å giving a geometry that is typical of Cu(I) complex tetrahedral geometries formed by interaction with the lone pair ligand electrons in sp3 hybrid orbitals on the metal. The two additional metal-ligand interactions are not typical, being nitrogen instead of a sulphur as the third equatorial copper coordinating ligand (Type I centres), or lacking a third histidine as in Cu_B_ copper centres. Interaction distances are about 2.5±0.14 Å for the water oxygen atom and about 2.9±0.09 Å for the N1 atom of Kyn62. The distance of the copper ion and the nitrogen atom of the kynurenine (2.9 Å) is considerably longer than what is usually considered a copper-nitrogen interaction [51], [52]. The kynurenine is, therefore, not a first-sphere ligand, but the ring amino group is close enough to be a second sphere copper ligand. The Ca^2+^ ion is bound in a somewhat distorted octahedral geometry with ligand-metal-ligand angles near 90 degrees and with metal-ligand bond distances of 2.3 to 2.4 Å. This geometry employs only mono-dentate acid side chain ligands that are typical of non-regulatory calcium binding sites that exhibit similar binding geometries whether magnesium or calcium is bound [53]. Our presumption is that the conserved Ca^2+^ binding site is important for the stabilization of the structures of these Cu(I) binding proteins.

A feature of the CorA crystal structure, which is not found in MopE*, is the presence of four ordered polyethylene glycol (PEG) molecules bound at the protein surface (Figs. 6c and S5). It was also observed in the crystal structure that Met57 in CorA might have been modified: a significant loss of electron density was observed for the methylthio group of this methionine. At the same time, significant difference in electron density was observed very close to the main chain atoms between Met57 and the following residue (Gly58). Flipping of the peptide bond was tried, but the electron density resembled more a cyclisation of the side chain. The change in electron density of Met57 suggested a change in mass. We therefore used MS for further analysis. However, we were not able to obtain proteolytic fragments from neither rec-CorA nor CorA that contained unmodified or modified Met57 and/or the modified Trp62 (Kyn62). The lack of MS data prevents us from concluding whether the unusual electron density at residue 57 is a disorder or a modification which has taken place *in vivo* prior to crystallization or is a result of crystallization or of radiation damage [54]. However, as presented above, the electron density clearly showed a modification of residue 62 (Trp62 to Kyn62) (Fig. 6c).

## Discussion

In the present work, we have purified to homogeneity the MopE homologous protein, CorA, from *M. album* BG8 and rec-CorA expressed in *E. coli*. Following purification, metal binding analyses using ICP-MS showed that only endogenous CorA contained one copper atom per protein molecule, and XANES analysis indicated that this copper atom is in its reduced (Cu(I)) state. Photoreduction of Cu(II) to Cu(I) in CorA by the X-rays is not considered to be very likely based on analogy to the MopE*, where the XANES spectrum was measured in fluorescence mode to prevent photoreduction. Furthermore, we have solved the structure of endogenous CorA using X-ray crystallography. The structure of CorA has a fold similar to MopE*, and confirmed the presence of a mononuclear copper-binding site (Fig. 6c). The asymmetric unit of the CorA crystals consists of six molecules forming two trimers. Analysis of interaction surfaces between monomers using the PISA server (‘Protein interfaces, surfaces and assemblies’ service PISA at the European Bioinformatics Institute (http://www.ebi.ac.uk/pdbe/prot_int/pistart.html) gives buried surface area of around 700 Å^2^ [55]. This suggests that the trimerization may be a crystallographic artefact rather than a biological entity. NaCl extraction of both whole cells and isolated outer membranes provided evidence that CorA is non-covalently associated to the *M. album* BG8 cellular surface. This cellular localization is in contrast to the previously assumed location of CorA to the inner membranes, but in line with the cellular localization determined for its homologs MopE and MEALZv2_1030034 proteins [11], [20], [24].

It is interesting to note the presence of 3 molecules of PEG bound to the surface of each of the CorA monomers in the crystal structure (Fig. 6a, Fig. S5), with two of the PEG molecule bound at interfaces between two adjacent CorA monomers. The significance of these bound PEG molecules is not understood, but their binding sites might point at binding sites for other ligands or co-factors. It should be noted that ordered PEG molecules could not have been observed in the MopE structure because the crystals of MopE were grown using ammonium sulfate as a precipitant [18].

CorA has previously been suggested to be involved in the *M. album* BG8 copper homeostasis. Moreover, a *corA* knock-out mutant grew very poorly and were not able to sustain growth even at higher copper concentrations, suggesting that CorA has a vital physiological role, putatively in the handling/transport of copper ions into the cells [23]. However, the inability to rescue the cells with copper made it difficult to draw any conclusions concerning the exact role of CorA in copper uptake or copper processing (ibid.). A homologous protein of CorA, MEALZv2_1030034, was recently isolated from the cell surface of another Type I methanotroph, the halotolerant *M. alcaliphilum* 20Z [20]. A constructed *M. alcaliphilum* 20Z mutant defective in the *MEALZv2_1030034* gene lost its ability to grow on methane. The growth of the *M. alcaliphilum* 20Z *MEALZv2_1030034* mutant could be rescued by adding methanol to the growth medium, thus bypassing the initial copper-dependent step in the methanotrophic metabolism (ibid.). The *MEALZv2_1030034* mutant phenotype is consistent with a role of MEALZv2_1030034 in the acquisition of copper ions for delivery to the *M. alcaliphilum* 20Z pMMO, equivalent to the suggested physiological function of CorA. Moreover, our findings substantiate that CorA and its homologous proteins MopE and MEALZv2_1030034 play an active role in the copper handling/uptake, which again is necessary for providing copper to pMMO required for its methane monooxygenase enzymatic activity.

The protein structure of CorA revealed in detail the structural properties of the copper-binding site (Fig. 6c). The copper atom is coordinated in a mononuclear binding site via two histidines, one water-molecule, and the tryptophan metabolite, kynurenine. This arrangement is analogous to the copper-binding site identified in MopE*, and emphasizes the importance of kynurenine as a copper binding ligand in these proteins [18]. The residues constituting the MopE/CorA copper-binding site are also conserved in the homologous MEALZv2_1030034 protein, strongly suggesting that MEALZv2_1030034 binds copper in an identical manner (Fig. S1). Furthermore, *CorA* is co-transcribed with a c-type cytochrome (*CorB*) that shares sequence similarity with members of the bacterial di-heme cytochrome c peroxidases (BCCP) [28]. A di-heme cytochrome c peroxidase with high sequence identity to CorB is also co-transcribed with *mopE* in *M. capsulatus* Bath (SACCP), and an equivalent c-type heme protein appear to constitute a gene locus with *MEALZv2_1030034* in *M. alcaliphilum* (MEALZv2_1030035) [11], [20]. Both CorB and MEALZv2_1030035 appear to be predominantly located in the periplasm, while SACCP is co-localized with MopE on the *M. capsulatus* Bath cellular surface (ibid.). Included in the BCCP protein family are the MauG proteins that are present in several facultative methylotrophs. MauG knockout mutants have demonstrated that these proteins are responsible for the formation of the tryptophan-trypthophyl quinone cofactor in methylamine dehydrogenase, a modification that involves the crosslinking of two tryptophans and is a prerequisite for its enzymatic activity [56], [57]. The resemblance of CorB and SACCP to MauG proteins, and the oxidation of tryptophan to kynurenine in both CorA and MopE, suggest that these endogenous c-type cytochromes are involved in the oxidation of tryptophan to kynurenine, the latter being important for binding of copper in this site [18]. Further work is required to characterize the copper-binding abilities of MEALZv2_1030034. However, it was shown that a *M. alcaliphilum* 20Z MEALZv2_1030035 knock-out mutant exhibited an increased growth rate on methane compared to the wild type cells, and had the ability to grow in more alkaline growth medium (pH 11) [20]. These findings suggest that MEALZv2_1030034 still was able to bind copper without the involvement of the di-heme cytochrome c peroxidase. Schukin et al. suggest that the increased activity of C_1_ oxidation enzymes in alkaline medium promote formation of reactive oxygen species that may lead to a non-enzymatic oxidation of tryptophan to kynurenine in the MEALZv2_1030034 putative copper-binding site in the absence of MEALZv2_1030035 [20]. However, the exact biological roles of the di-heme cytochrome c peroxidases in regard to the biological role of CorA and its homologs remain to be elucidated.

Data supporting the role of methanobactins in copper acquisition and copper handling in (many) methanotrophs are rapidly emerging [3], [17]. Interestingly, it has been demonstrated that methanobactins isolated from *M. album* BG8 and *M. capsulatus* (Bath) have a much lower binding affinity to copper compared to the methanobactin isolated from the alpha-proteobacterium (Type II) *Methylosinus trichosporium* OB3b [58], [59]. On the other hand, the copper-binding affinity for MopE* is in the high affinity range as observed for the methanobactin from *M. trichosporium* OB3b. The use of Tris in the gelfiltration buffers and storage buffers can also provide some indications regarding the affinity of Cu(I) for CorA. Tris is a Cu(II)-chelator [60], [61], and excess of Tris reacts with Cu(II) to yield complexes with cumulative formation constants β4 = 5.5×10^13^ [62], [63]. The small amount of Cu(II) present in the buffer as impurities, and the even smaller amount of Cu(I) present (which under the oxidizing conditions used will be oxidized to Cu(II)), is therefore bound as Cu-Tris-complex(es). The same should also inevitably be the fate of any Cu(I) lost from CorA in the same buffer. CorA is being stored in 40 mM Tris-HCl, pH 8.0, 0.5 M NaCl, after gelfiltration in the same buffer, without any apparent change in its 1∶1 content of Cu(I). This can only be explained by an apparent binding constant lower than 10^−12^, which is comparable to those found by various methods for several other copper(I) binding affinities, as recently discussed by Xiao et al. [64]. Moreover, proteins similar to CorA and MopE appear not to be present in *M. trichosporium* OB3b and *Methylobacter tundripaludum* SV96 (Type I) genomes [65], [66]. These latter observations suggest that, although these proteins may be linked to the activity of pMMO, their presence is not directly related to the synthesis of pMMO. This is in line with the finding that *pmoA* is constitutively expressed in *M. capsulatus* Bath [6]. It has been suggested that the presence of CorA homologues in *M. album* BG8, *M. capsulatus* Bath, and *M. alcaliphilum* 20Z may be related to the formation of S-layers in these bacteria, which may necessitate mechanisms for copper acquisition other than methanobactins [20].

The present work provides additional evidence for a significant role of CorA, MopE, and the MEALZv2_1030034 protein as part of a two-gene copper acquisition system present in these (divergent) group of methanotrophic bacteria, utilizing both chalkophores and copper binding surface associated proteins in a high-affinity copper uptake strategy. This would appear as advantageous among methanotrophic communities experiencing very diverse environmental conditions, in particular regarding copper availability.

## Supporting Information

Figure S1 **Structural alignment of** ***M. album*** **BG8 CorA,** ***M. alcaliphilum*** **20Z MEALZv2_1030034 and** ***M. capsulatus*** MopE*. The structure based sequence alignment of CorA, MopE* and MEALZv2_1030034 was generated by combining a structural alignment of CorA and MopE* generated by the Dali server (http://ekhidna.biocenter.helsinki.fi/dali_lite/) (44) and a standard sequence alignment of CorA and MEALZv2_1030034 using Clustal X. The figure is generated using the ESPript server (espript.ibcp.fr). Secondary structure elements, including numbering of the elements, of CorA and MopE* are marked above and under the alignment, respectively. Blue stars indicate residues involved in copper binding, blue triangles indicate residues involved in calcium binding and blue bars indicate residues close to PEG molecules (generally between 3.4 and 4.0 Å).
(TIF)

Figure S2 **Packing of the 6 CorA molecules found in the asymmetric cell of the CorA crystal.** a) Side view, and b) rotated 90° around the horizontal axis.
(TIF)

Figure S3 **SDS-PAGE assessment of the purified recombinantly expressed CorA protein (rec-CorA).**
(TIF)

Figure S4 **Circular dicroism analysis of purified rec-CorA.** rec-CorA in 20 mM Potassium phosphate buffer pH 7.5.
(TIF)

Figure S5 Electrostatic surface of CorA (left) and MopE* (right) illustrating the copper binding sites (yellow spheres) and PEG molecules (ball-and-stick models) found on the CorA surface. The electrostatic potential is contoured from −10 (red) to +10 (blue) kT/q. The copper binding sites in CorA and MopE* are in the same orientation.
(TIF)
