# Supplementary figures and images for: CorA Is a Copper Repressible Surface-Associated Copper(I)-Binding Protein Produced in Methylomicrobium album BG8
Source: PLoS One. 2014 Feb 3;9(2):e87750. doi: 10.1371/journal.pone.0087750 (PMC3912023; doi:10.1371/journal.pone.0087750)

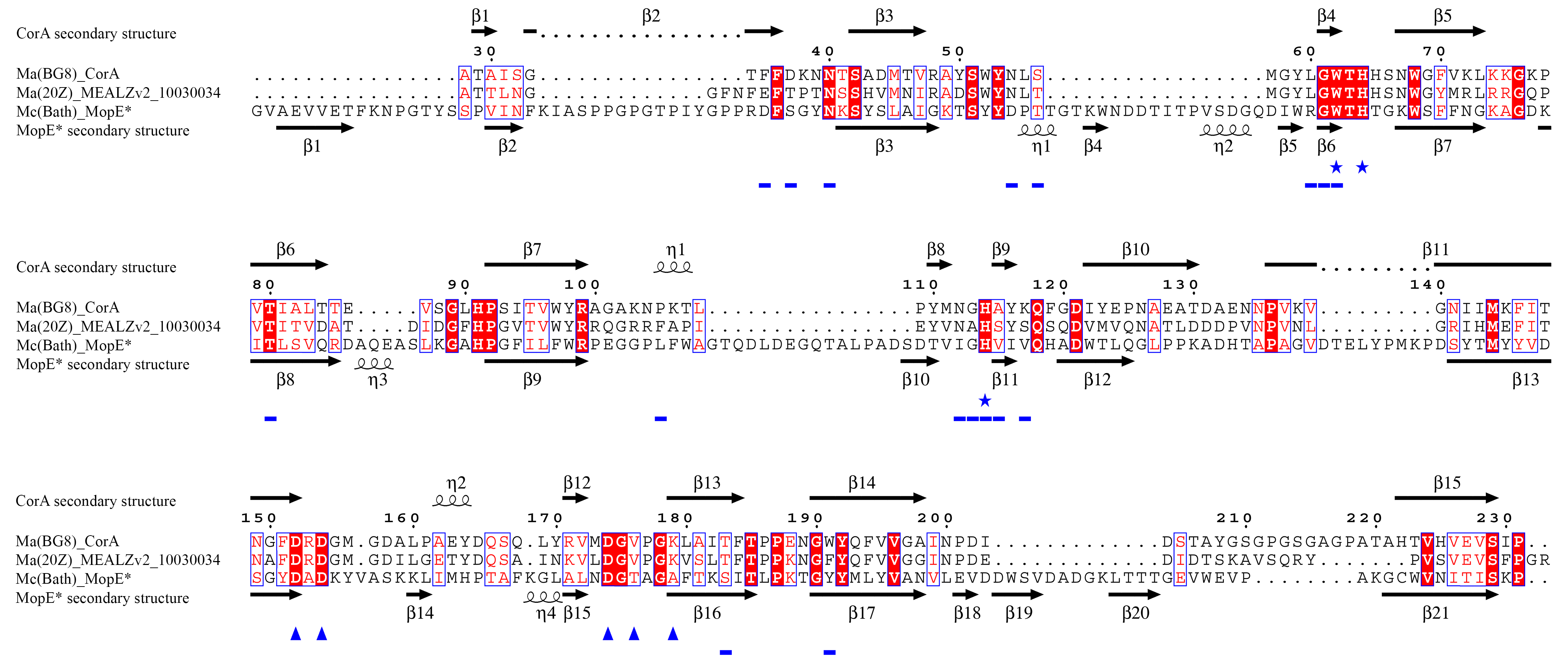

Supplement: Figure S1 — Structural alignment of M. album BG8 CorA, M. alcaliphilum 20Z MEALZv2_1030034 and M. capsulatus MopE*. The structure based sequence alignment of CorA, MopE* and MEALZv2_1030034 was generated by combining a structural alignment of CorA and MopE* generated by the Dali server (http://ekhidna.biocenter.helsinki.fi/dali_lite/) (44) and a standard sequence alignment of CorA and MEALZv2_1030034 using Clustal X. The figure is generated using the ESPript server (espript.ibcp.fr). Secondary structure elements, including numbering of the elements, of CorA and MopE* are marked above and under the alignment, respectively. Blue stars indicate residues involved in copper binding, blue triangles indicate residues involved in calcium binding and blue bars indicate residues close to PEG molecules (generally between 3.4 and 4.0 Å). (TIF) [file pone.0087750.s001.tif]

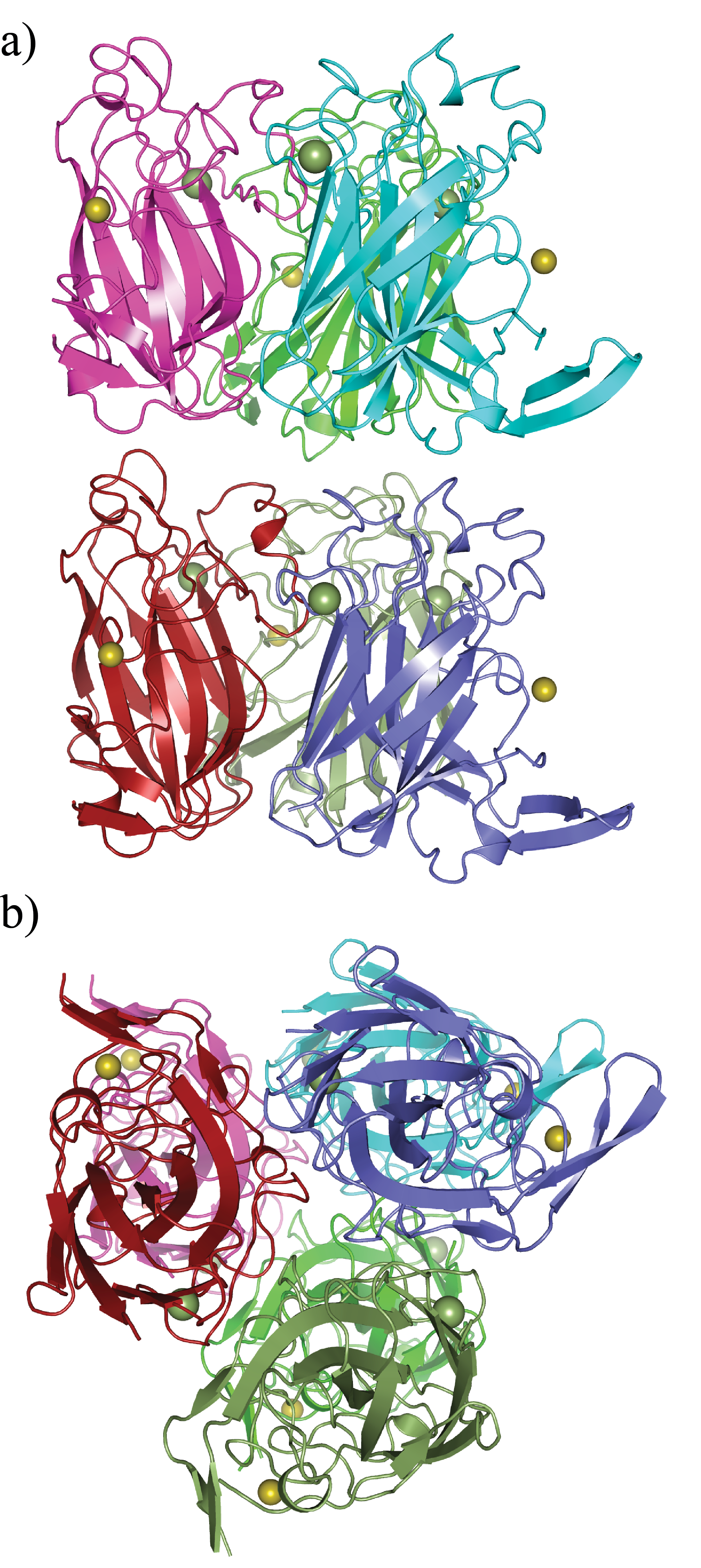

Supplement: Figure S2 — Packing of the 6 CorA molecules found in the asymmetric cell of the CorA crystal. a) Side view, and b) rotated 90° around the horizontal axis. (TIF) [file pone.0087750.s002.tif]

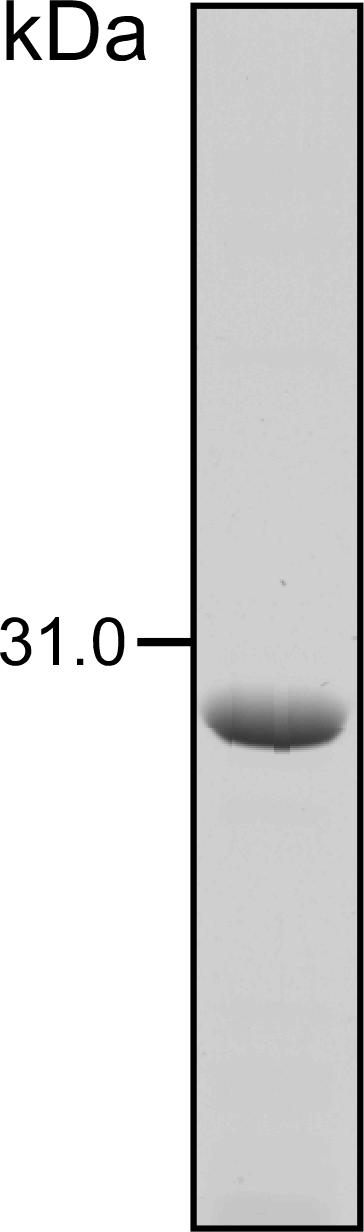

Supplement: Figure S3 — SDS-PAGE assessment of the purified recombinantly expressed CorA protein (rec-CorA). (TIF) [file pone.0087750.s003.tif]

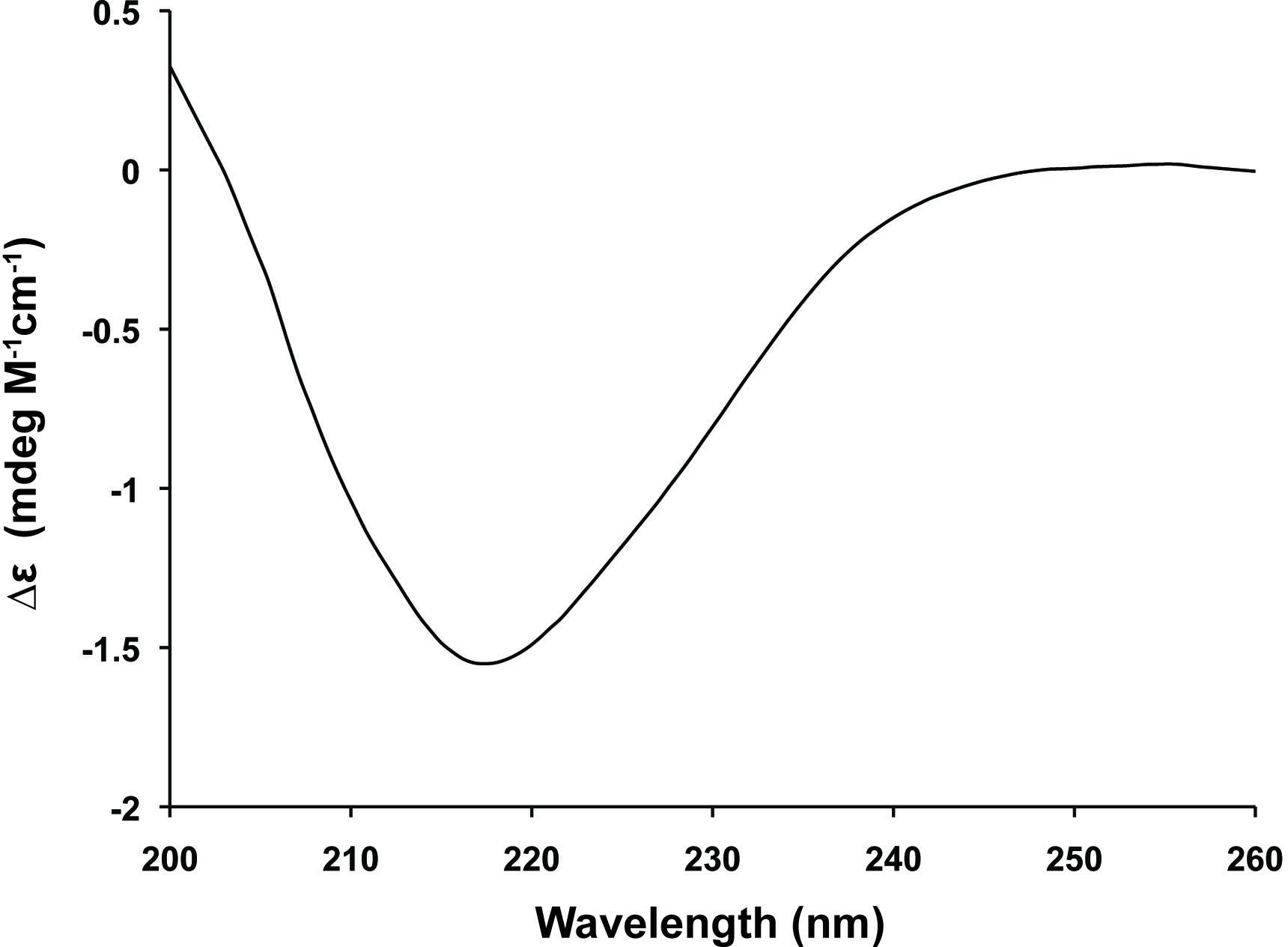

Supplement: Figure S4 — Circular dicroism analysis of purified rec-CorA. rec-CorA in 20 mM Potassium phosphate buffer pH 7.5. (TIF) [file pone.0087750.s004.tif]

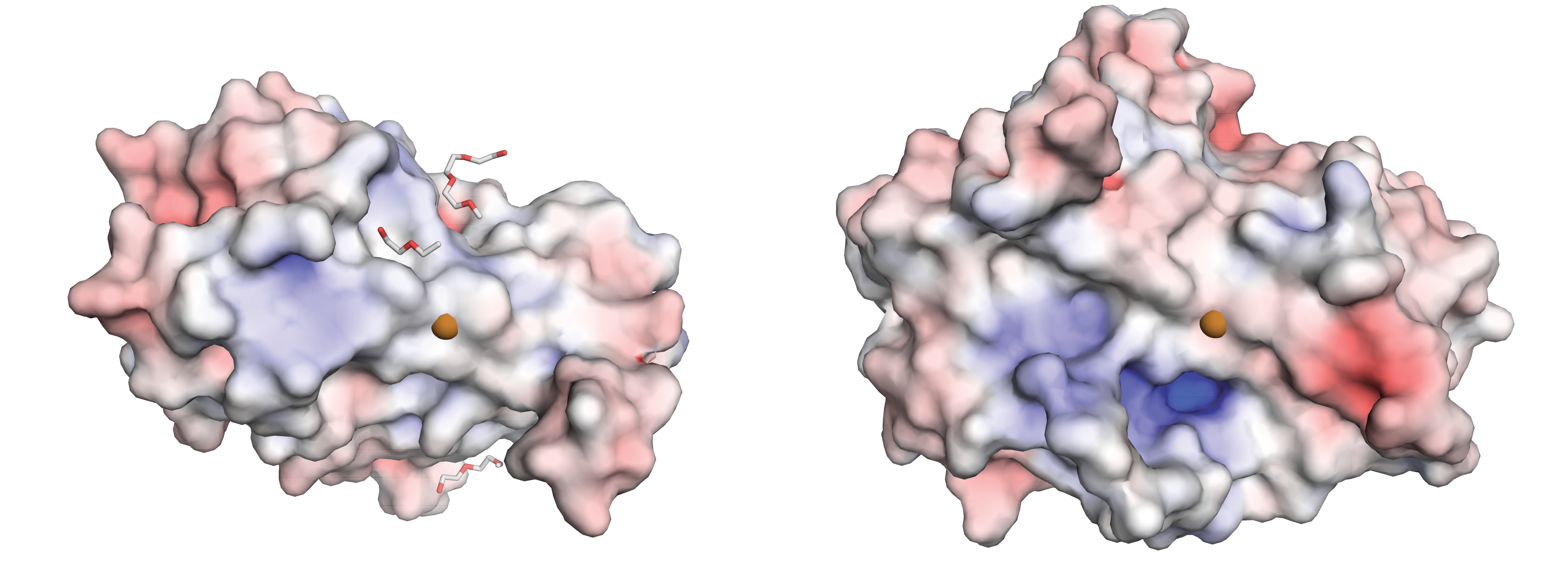

Supplement: Figure S5 — Electrostatic surface of CorA (left) and MopE* (right) illustrating the copper binding sites (yellow spheres) and PEG molecules (ball-and-stick models) found on the CorA surface. The electrostatic potential is contoured from −10 (red) to +10 (blue) kT/q. The copper binding sites in CorA and MopE* are in the same orientation. (TIF) [file pone.0087750.s005.tif]
